# Bizarre Parosteal Osteochondromatous Proliferation (Nora's Lesion) of the Foot: A Case Report and Literature Review of Nora's Lesion of the Foot

**DOI:** 10.7759/cureus.24197

**Published:** 2022-04-17

**Authors:** Koray Başdelioğlu, Ayşe Nur Toksöz Yıldırım, Krishna Reddy, Korhan Özkan

**Affiliations:** 1 Orthopaedics and Traumatology, Yeditepe University Faculty of Medicine, Istanbul, TUR; 2 Pathology, Istanbul Medeniyet University Göztepe Training and Research Hospital, Istanbul, TUR; 3 Orthopaedics and Traumatology, University of Cincinnati Medical Center, Cincinnati, USA; 4 Orthopaedics and Traumatology, Istanbul Medeniyet University Göztepe Training and Research Hospital, Istanbul, TUR

**Keywords:** parosteal osteosarcoma, periosteal chondroma, foot, nora’s lesion, bizarre parosteal osteochondromatous proliferation

## Abstract

Bizarre parosteal osteochondromatous proliferation (BPOP) or Nora's lesion is a rare, benign, but locally aggressive tumor. We present a case of a 45-year-old patient with progressive swelling of his toe for four years, pain, and difficulty with shoe wear. The lesion was excised after adequate evaluation and the resection histopathology was compatible with Nora’s lesion. There was no local recurrence at 24 months of follow-up. Nora's lesion can be effectively treated by complete surgical excision or en bloc resection. Though rare, Nora's lesion should be considered in the differential diagnoses of osteogenic and/or chondrogenic overgrowths in the bones of feet such as subungual exocytosis, osteochondroma, chondrosarcoma, periosteal chondroma/chondrosarcoma, and parosteal osteosarcoma.

## Introduction

Bizarre parosteal osteochondromatous proliferation (BPOP) or Nora's lesion is a rare, benign, but locally aggressive tumor described by Nora et al. in 1983 [1]. Nora's lesion mostly affects the hands and feet but appears four times more commonly in the hands than the feet [2]. It affects women and men equally and is mostly seen between the ages of 20 and 30 years [3]. The lesion is characterized by well-circumscribed exophytic overgrowth of the periosteal region with intact cortex without any medullary changes [4]. Although it shows a benign character on imaging, it is a challenge to distinguish it from malignant tumors such as chondrosarcoma and parosteal osteosarcoma due to its aggressive characteristics and complex histopathological features [2-5].

In this report, we present a case of Nora’s lesion in the middle phalanx of the second toe with characteristic imaging and histological features. We review the literature and discuss the diagnostic dilemma for orthopedic oncologists, radiologists, and pathologists.

## Case presentation

A 45-year-old male patient presented to our outpatient clinic with a complaint of swelling in the left second toe. He was aware of this gradually increasing swelling for about 18 months and more recent history of pain and difficulty with shoe wear. His symptoms were made worse with walking/activity and he did not have any rest pain. There was no history of trauma that the patient could recollect. On physical examination, a hard, immobile, 10 x 10 mm mass was detected in the middle phalanx of the second toe. There was no dermal lesion except erythema due to the compression effect of the mass during shoe wear.

**Figure 1:**
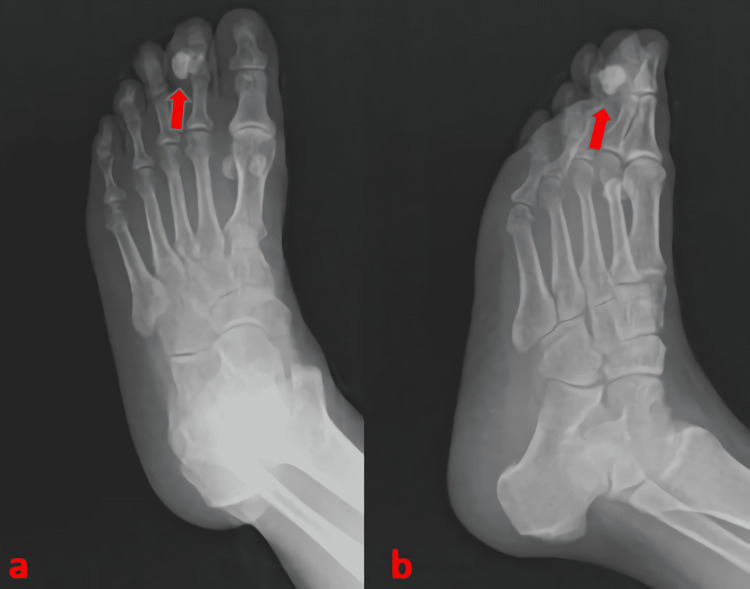
Preoperative X-ray images.

The patient’s blood laboratory workup was normal. Radiographs of the foot showed exostosis with irregular bone density in the middle phalanx of the second toe. The mass had no connection or continuity with the medullary cavity (Figure 1).

**Figure 2:**
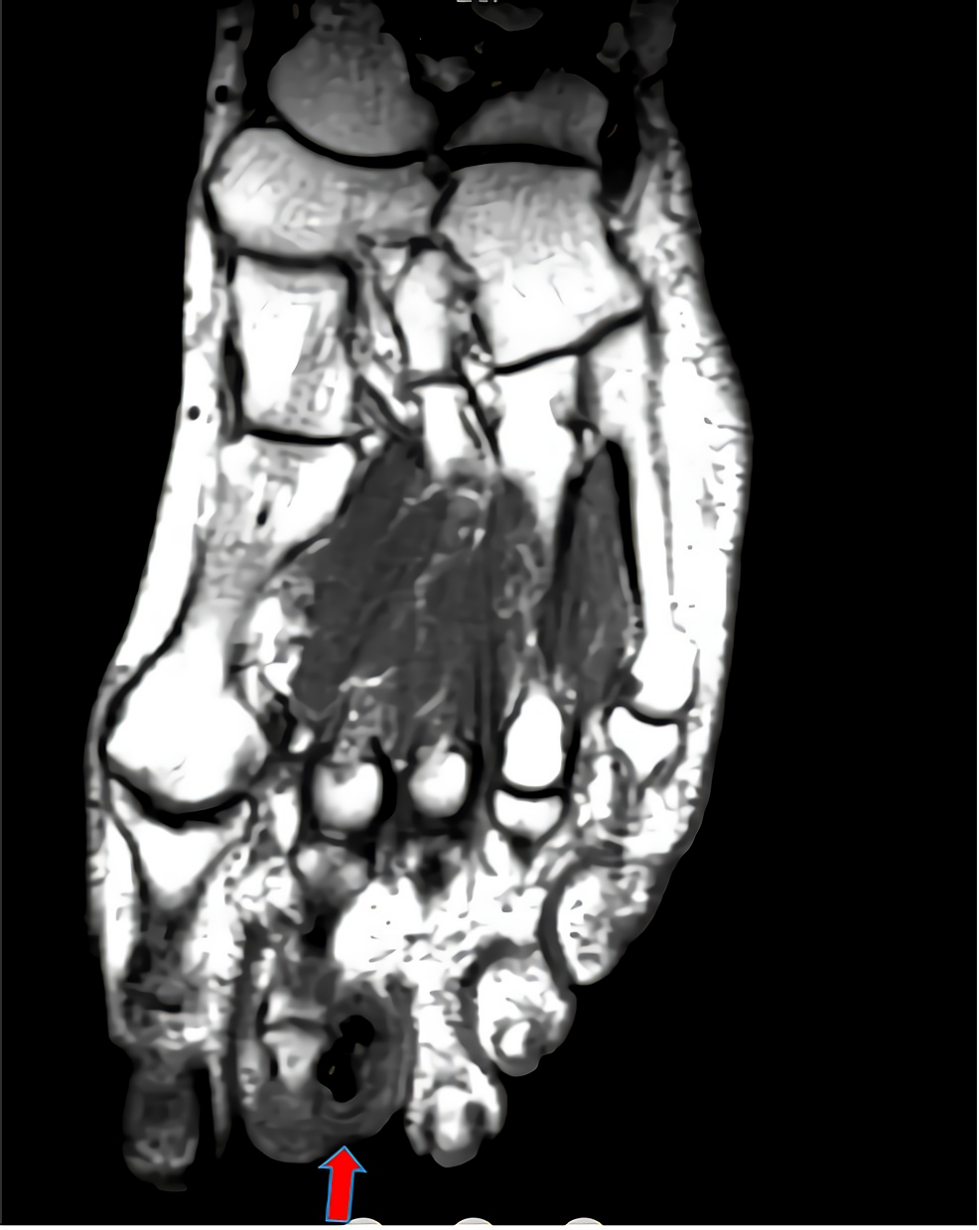
A hypointense lesion was seen on MRI.

MRI with and without contrast was performed. MRI displayed a 13.6 x 8 mm, T1-T2 hypointense lesion with no contrast enhancement or soft tissue component (Figure 2).

**Figure 3:**
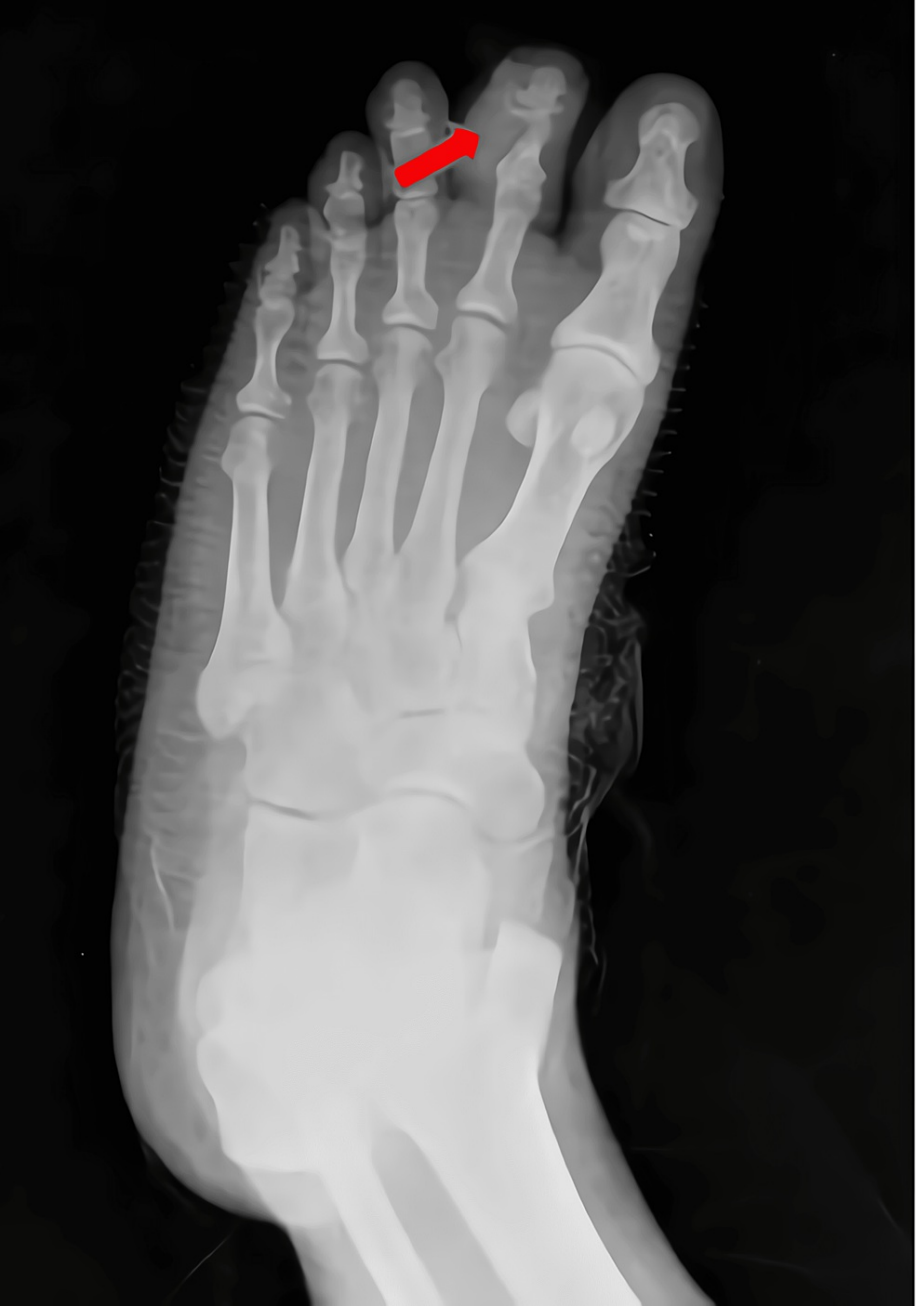
Postoperative X-ray image.

An excisional biopsy of the lesion was carried out with clear margins. The neurovascular structures were preserved. A small part of the cortex was excised as part of the planned margin, bearing in mind the possibility of parosteal osteosarcoma or periosteal chondrosarcoma. A postoperative X-ray image is shown in Figure 3.

**Figure 4:**
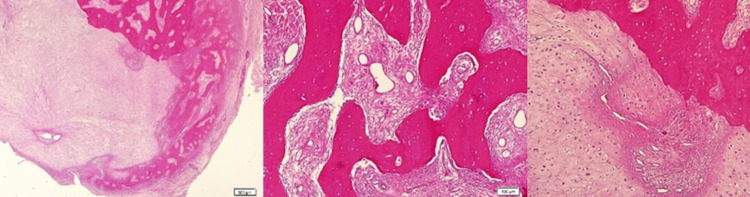
Irregular bone trabeculae surrounded by active osteoblasts and one-half of the lesion showed an island consisting of chondrocytes with enlarged nuclei and binucleation were observed.

Histopathology of the excised lesion showed that the mass was covered by a thin fibrous capsule in most areas. In cross-sections, immature irregular bone trabeculae surrounded by active osteoblasts were observed in a loose fibrous stroma consisting of spindle cells. One-half of the lesion showed an island consisting of chondrocytes with enlarged nuclei and binucleation was observed (Figure 4).

**Figure 5:**
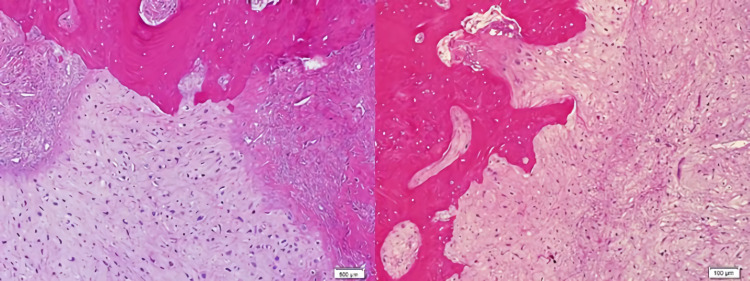
The cartilaginous tissue eroded bone in focal areas.

Marked hyperchromasia and atypical mitoses were not seen. The cartilaginous tissue eroded bone in focal areas (Figure 5).

In the light of these findings, a diagnosis of BPOP (Nora's lesion) was made histopathologically. No complications were encountered during or after the surgery. The patient was allowed to weight bear on his foot the next day after the surgical procedure. At 24 months postoperatively, the patient has continued to be pain-free with full restoration of his daily activities without any problem. The patient was informed that data concerning the case would be submitted for publication and the patient gave her approval.

## Discussion

Literature review

A literature search was carried out on PubMed, ScienceDirect, EBSCO Discovery Service, Embase, Scopus, Medscape, and Google Scholar electronic databases using the keywords “Nora's lesion,” “Bizarre Parosteal Osteochondromatous Proliferation,” and “foot.” We tailored our search to those in the English language only. There were 26 studies reporting Nora's lesion of the foot. Studies with missing data were excluded and 14 case reports were evaluated. All studies were analyzed from past to present according to the criteria of age, gender, localization, treatment, and outcomes. The results are shown in Table 1.

**Table 1:** Details of the patients with Nora's lesion of the foot in the literature.

Case	Author	Year	Age/gender	Localization	Side	Treatment	Follow-up (month)	Outcome
1	Bandiera et al. [6]	1998	47/M	Proximal phalanx of the great toe	R	Excision	19	No recurrence
2	Harty et al. [7]	2000	32/F	First metatarsophalangeal joint	L	Excision	12	No recurrence
3	Saygi et al. [8]	2006	19/F	Proximal phalanx of the fifth toe	L	Excision	30	No recurrence
4	Rybak et al. [9]	2007	15/F	Fourth metatarsal	R	Excision	6	Recurrence
5	Boussouga et al. [10]	2008	42/M	Fifth metatarsal and proximal phalanx of the fifth toe	L	Excision	24	No recurrence
6	Teoh et al. [11]	2009	12/M	Second metatarsal	R	Excision	12	No recurrence
7	Singh et al. [12]	2010	65/F	Whole phalanx of the second toe	L	Excision	36	No recurrence
8	Suresh [13]	2010	17/M	Proximal phalanx of the second toe	R	Excision	48	No recurrence
9	James and Henderson [14]	2013	10/M	Distal phalanx of the great toe	R	Excision	18	Multiple recurrences
10	Doganavsargil et al. [15]	2014	24/F	Second metatarsal	L	Excision	48	No recurrence
11	Nayak et al. [16]	2017	51/M	Proximal and distal phalanx of the great toe	L	Excision	14	Recurrence
12	Rushing et al. [17]	2017	48/F	Calcaneus	R	Surgery was refused by the patient after the biopsy	18	No symptom
13	Bajwa et al. [5]	2019	36/F	Fourth metatarsal	R	Excision	6	Recurrence
14	Yao et al. [18]	2020	57/F	Third metatarsal	R	Excision	12	No recurrence
15	Present case	2021	45/M	Middle phalanx of the second toe	L	Excision	24	No recurrence
Summary (excluding present case)			Age: 33.9 (10-65), gender: 6M/8F	Great toe - proximal phalanx: 1; distal phalanx: 1; proximal and distal phalanx: 1; metatarsophalangeal joint: 1. Second toe - proximal phalanx: 1; proximal/middle/distal phalanx: 1. Fifth toe - proximal phalanx: 2. Metatarsals - second: 2; third: 1; fourth: 2; fifth: 1. Calcaneus: 1	8R/6L	13 excision/1 refusion after biopsy	Mean follow-up: 20.3 (6-48)	No recurrence: 10, recurrence: 3

Epidemiology

Nora's lesion is a rare, benign, but locally aggressive tumor characterized by overgrowth of the periosteal surface [1,4]. It affects both genders equally and is more common between the ages of 20 and 30 years. The age range of Nora's lesion of the foot varies between 10 and 65 years with a mean age of 33.9 years. Eight of these lesions were in females and the remaining six in males (Table 1). Nora's lesion commonly affects the hands four times as often as the foot [2]. The distribution of Nora's lesion seen in the forefoot in the literature is shown in Table 2. There were no reported cases of involvement of the middle phalanx. In addition, there was one such lesion in the calcaneus.

**Table 2:** The distribution of Nora's lesion seen in the forefoot.

Site	Proximal	Distal	Metatarsal	Combined	Total
Great toe	1	1	1	1	4
Second toe	1		2	1	4
Third toe			1		1
Fourth toe			2		2
Fifth toe	2		1		3

Clinical features

Patients with Nora's lesion usually present with a mass that grows slowly over weeks to months and may or may not cause any pain [1]. Some theories have been presented in terms of the etiopathogenesis of Nora's lesion [19,20]. Meneses et al. reported that previous trauma may be an effective factor in the formation of Nora's lesion [21]. Cytogenetic studies reported some translocations with the commonest being t(1;17)(q32;q21) and other variant translocations involving 1q32 may be important in the development of Nora's lesion [19].

Radiologic features

On plain radiographs, it appears as a well-circumscribed mass that arises from the bone surface and is attached to the underlying cortex, and may be protected by soft tissue [21]. While X-rays provide significant useful information, the MRI is much more effective in showing the characteristic of Nora's lesion. MRI also provides important clues in the differential diagnosis. Intact cortex and absence of soft tissue swelling and medullary involvement are important clues in favor of Nora's lesion [22,23]. BPOP lesions can be seen on MRI images as a low signal in T1-weighted sequences and as low to medium signal in T2-weighted images. Additionally, depending on the degree of calcification/ossification, short tau inversion recovery (STIR) imaging may show low or high signal characteristics [22]. Computed tomography is the most helpful imaging method that distinguishes Nora's lesion from osteochondroma. The lack of continuity with the adjacent bone and medullary bone and the absence of a cartilage cap are important features in differential diagnosis [22,23]. In the present case report, classical radiological features of Nora's lesion described in the literature were seen.

Histopathology

Histologically, Nora's lesion consists of three different structures: bone, cartilage, and spindle cells [24]. On gross examination, the lesion has a nodular surface covered with cartilage and a blue hue inside the bone [21]. It has a heterogeneous structure randomly formed of cartilage tissue, bone, and proliferative fibroblasts. There are well-defined lobulated cartilage cells around the lesion. This structure may be accompanied by a fibrous pseudocapsule. In Nora's lesion, enchondral ossification is irregular with limited organization and areas of irregular ossification. The calcified matrix has a blue tint and hence is also called “blue bone,” a characteristic of Nora's lesion. While minimal cytological atypia is seen in proliferative spindle fibroblasts and osteoblasts in bony trabeculae, cartilage is usually hypercellular and chondrocytes are enlarged [21,25,26].

The differential diagnosis of Nora's lesion includes subungual exocytosis, osteochondroma, chondrosarcoma, periosteal chondroma, and parosteal osteosarcoma [2,25,26]. These situations should also be considered during histopathological examination.

Treatment and prognosis

Definitive treatment of Nora's lesion includes surgical excision of the lesion and curettage of the base of the lesion after excision [1,21]. Local recurrences have been reported with most occurring within the first two years [1,23]. Based on the review of literature, we found the recurrence rate to be 23% (no recurrence = 10, recurrence = 3; Table 1).

## Conclusions

Diagnosis and treatment of Nora's lesion are challenging due to its rare occurrence. Although only a few cases of Nora's lesion have been reported, it should be considered in the differential diagnosis of osteogenic and chondrogenic overgrowths in the bones of the foot. Complete surgical excision of Nora's lesion is the most effective and beneficial method of treatment. Subungual exocytosis, osteochondroma, chondrosarcoma, periosteal chondroma/chondrosarcoma, and parosteal osteosarcoma should also be kept in mind in the differential diagnosis of Nora's lesion. Careful clinical, radiological, and histopathological evaluation assists with a definitive diagnosis and distinguishing it from other conditions listed in the differential diagnosis.
